# Establishment of a diverse pheno-genotypic challenge set of *Klebsiella pneumoniae* and *Pseudomonas aeruginosa* suitable for use in the murine pneumonia model

**DOI:** 10.1093/jac/dkae388

**Published:** 2024-10-30

**Authors:** Andrew J Fratoni, Alissa M Padgett, Erin M Duffy, David P Nicolau

**Affiliations:** Center for Anti-Infective Research and Development, Hartford Hospital, 80 Seymour Street, Hartford, CT 06102, USA; Center for Anti-Infective Research and Development, Hartford Hospital, 80 Seymour Street, Hartford, CT 06102, USA; CARB-X, Boston, MA, USA; Center for Anti-Infective Research and Development, Hartford Hospital, 80 Seymour Street, Hartford, CT 06102, USA

## Abstract

**Background:**

Preclinical murine infection models lack inter-laboratory uniformity, complicating result comparisons and data reproducibility. The European Innovative Medicines initiative-funded consortium (COMBINE) has developed a standardized murine neutropenic pneumonia protocol to address these concerns. While model methods have been standardized, a major obstacle to consistent results is the lack of available bacteria with defined viability and variability. Herein, we establish a diverse challenge set of *Klebsiella pneumoniae* and *Pseudomonas aeruginosa* suitable for use in the COMBINE protocol to further minimize experimental inconsistency and improve the interpretability of data generated among differing laboratories.

**Materials and methods:**

Sixty-six *K. pneumoniae* and 65 *P. aeruginosa* were phenotypically profiled against tigecycline (*K. pneumoniae* only), levofloxacin, meropenem, cefiderocol and tobramycin. Fifty-nine isolates were introduced into the COMBINE model to assess the sufficiency of the starting bacterial inoculation, resultant baseline bacterial burden, achievement of ≥1 log_10_cfu/lung growth at 24 h, time to and percentage mortality. Forty-five isolates displaying desirable minimum inhibitory concentration profiles were subjected to replicate *in vivo* testing to assess target parameters.

**Results:**

83% of *K. pneumoniae* reached the prerequisite growth at 24 h using a starting bacterial burden ≥7 log_10_cfu/lung. *P. aeruginosa* isolates grew well in the model: 90% achieved the growth target with a starting bacterial burden of 6 log_10_cfu/lung. Mortality was negligible for *K. pneumoniae* but high for *P. aeruginosa*. Poor or inconsistent achievement of the 24 h growth target was seen in 11/59 isolates.

**Conclusions:**

With this diverse cache of viable isolates established in the COMBINE pneumonia model, future translational studies can be undertaken to set efficacy benchmarks among laboratories.

## Background

Preclinical murine infection models play an integral role in the development of direct-acting antibiotics by assessing efficacy and informing optimized dosing regimens through pharmacokinetic and pharmacodynamic analyses.^1–3^ Despite decades of use in supporting drug development, there remains a lack of uniformity in murine models that makes comparisons of results and data reproducibility problematic, calling into question clinical translation.^4–7^ While on the surface the historic use of *in vivo* murine pneumonia models may seem more similar than divergent, numerous variables often differ regarding the animals, bacteria, infection procedure, treatment and endpoints. Animals may have differences in strain, sex, age, weight and number per treatment group. The differing phenotypic and genotypic profiles and intrinsic virulence of the bacteria as well as the method of inoculum preparation (i.e. growth stage, bacterial density) can substantially alter the effectiveness of the test agent. Infection procedures vary in immunosuppression, anaesthesia and infection route. Dissimilarities exist in treatment outcomes relating to the time to initiation of treatment, baseline bacterial density, *in vivo* growth characteristics of the bacteria, drug exposure (pharmacokinetic/pharmacodynamic, PK/PD) profile at target site and duration of therapy. Importantly for a pneumonia indication, antibiotic exposures at the target site of infection, pulmonary epithelial lining fluid (ELF), are not consistently characterized. Instead, plasma concentrations are measured as a surrogate with little consideration for interspecies differences in target site penetration between mouse and man.^8^

A standardized global protocol has been established by the Collaboration for prevention and treatment of MDR bacterial infections (COMBINE) consortium in hopes of harmonizing the methods utilized in the murine pneumonia model.^9^ While adherence to the COMBINE protocol will assist in narrowing the gap in inter-laboratory variability, a major impediment to consistency in results and subsequent interpretations among these *in vivo* studies is the lack of available bacteria with defined phenotypic and genotypic profiles that reproducibly induce infection under the stipulated constructs of the COMBINE protocol. The goal of the current investigation is to establish a diverse challenge set of *Klebsiella pneumoniae* and *Pseudomonas aeruginosa* suitable for use in the COMBINE protocol to further minimize experimental inconsistency and improve the interpretability of endpoint data such as quantitative cfu or pharmacodynamic profiling among differing laboratories.

## Materials and methods

### Antimicrobial agents

Analytical grade powders were acquired as follows for *in vitro* broth microdilution testing: tigecycline (Supelco, lot no. LRAD6890), levofloxacin (Sigma Aldrich, lot no. BCCF6845), meropenem (Supelco, lot no. LRAD1014) and tobramycin (Supelco, lot no. LRAC7886). For cefiderocol, commercial vials were reconstituted for *in vitro* broth microdilution testing (Shionogi, lot no. 0021).

### *Bacterial isolates and* in vitro *susceptibility testing*

*K. pneumoniae* and *P. aeruginosa* isolates were sourced from the isolate repository at the Center for Anti-Infective Research and Development (CAIRD) (Hartford, CT, USA), the CDC (Centers for Disease Control and Prevention) and FDA (Food and Drug Administration) Antibiotic Resistance Isolate Bank (CDC Bank) (Atlanta, GA, USA), the Paul Ehrlich Institute (PEI) (Berlin, Germany) and the Leibniz Institute (DSMZ) (Brunswick, Germany). These contemporary clinical isolates, including those from COMBINE consortium sites PEI and DSMZ, were selected to encompass a variety of genotypic and phenotypic profiles resulting in a wide distribution of minimum inhibitory concentrations (MICs) against tigecycline, levofloxacin, meropenem, cefiderocol and tobramycin. These five agents have been selected because they represent differing drug classes: tetracycline-derivative, fluoroquinolone, β-lactam, siderophore-conjugate and aminoglycoside. In addition to differing mechanisms of action/resistance, this set of compounds represents divergence in both their pharmacokinetic and pharmacodynamic profiles, thus serving as ideal benchmarking therapies for the future assessment of novel agents in this humanized translational model.

Before experimentation (both *in vitro* and *in vivo*), each isolate was sub-cultured twice on Trypticase soy agar with 5% sheep blood (Becton Dickinson and Co., Sparks, MD, USA) and incubated at 37°C for ∼16 h. In total, 66 *K. pneumoniae* and 65 *P. aeruginosa* isolates were tested by broth microdilution on separate days in triplicate or until a modal value was achieved in accordance with Clinical and Laboratory Standards Institute guidance before introduction into the murine neutropenic pneumonia model.^10^ Tigecycline was only tested against *K. pneumoniae* isolates due to its lack of activity against *P. aeruginosa*. *Enterococcus faecalis* 29212 (levofloxacin MIC range 0.25–2 mg/L; meropenem MIC range 2–8 mg/L) and *Escherichia coli* 25922 (tigecycline MIC range 0.03–0.25 mg/L; cefiderocol MIC range 0.06–0.5 mg/L; tobramycin MIC range 0.25–1 mg/L) were used as quality control strains on each day of study.^11^ Colony counts were performed from the control well of every replicate in accordance with CLSI methods.^10^ If the colony count and/or the QC was out of range, the MIC data were discarded and repeated.

### Ethics

Animals were maintained and used in accordance with National Research Council recommendations. The study protocol was reviewed and approved by the Institutional Animal Care and Use Committee at Hartford Hospital (Assurance no. A3185-01).

### Laboratory animals and the neutropenic pneumonia model

The model followed the COMBINE protocol with laboratory-specific detailed methods as follows.^9^ Specific pathogen free CD-1, female mice 6–8 weeks old were acquired from Charles River Laboratories, Inc. (Raleigh, NC, USA). All animals were allowed to acclimatize for 72 h before any study procedures and were housed in groups of six at controlled room temperature in HEPA-filtered cages (Innovive, San Diego, CA, USA). Study rooms were maintained with diurnal cycles (12 h light/12 h dark) and food and water were provided *ad libitum*.

A predictable degree of renal impairment was produced using 5 mg/kg of uranyl nitrate administered intraperitoneal on day −3.^12^ Bacterial colonies from the overnight culture plate were suspended in NS to a McFarland target of 2.5 and further diluted in saline to produce the final inoculum. Mice were anaesthetized using inhaled isoflurane, manually restrained upright and infected with 50 µL of bacterial suspension via the nares. Each bacterial inoculation suspension was used within 30 mins of initial preparation and was serially diluted and plated to confirm the cfu/mL.

Two hours after bacterial inoculation, 3–6 mice per isolate were sacrificed via CO_2_ asphyxiation followed by cervical dislocation to determine initial bacterial burden in the model. An additional six mice per isolate were euthanized 24 h later, or on earlier infection-related mortality or loss of the righting reflex. Lungs from each mouse were harvested aseptically and homogenized. Homogenates were serially diluted and plated on Trypticase soy agar with 5% sheep blood and incubated at 37°C for ∼16 h before cfu enumeration. Inoculum bacterial suspension cfu/mL, baseline bacterial burden cfu/lungs, cfu/lung at 24 h, time to and percentage mortality over the 24 h post-inoculation period before scheduled harvest time were quantified for each isolate.

### *Selection of isolates for initial and replicate* in vivo *testing and data analysis*

For consideration of inclusion in the *in vivo* testing system, a modal MIC value had to be established for each organism using the gold standard, broth microdilution technique. Isolates that displayed a >8-fold spread (i.e. three doubling dilutions) in the MIC distribution from the modal value were considered to have highly variable phenotypic profiles and were excluded from subsequent *in vivo* studies. Test isolates considered for replicate *in vivo* testing were required to display a consistent inter-mouse starting bacterial burden, defined as having all samples in the group within 1 log_10_ cfu/lung of each other, based on the quantitative assessment at 2 h after inoculation. Test isolates considered for replicate *in vivo* testing were also required to display sufficient growth at 24 h, defined as ≥1 log_10_ cfu/lung relative to the starting bacterial burden. Last, test isolates considered for replicate *in vivo* testing were also required to display limited infection-related mortality over the initial 8 h post-inoculation period as acute mortality due to overwhelming sepsis during this timeframe does not allow for the discrimination of drug related activity.

The sole purpose of this multi-month, repetitive assessment of isolates was to define the reproducibility of the target model parameters with the standardized employed COMBINE methods as reported. Therefore, the performance of each isolate was individually assessed based on its ability to reproducibly be recovered 2 h after intranasal inoculation and grow over the 24 h study period while not causing overt mortality in the initial hours after inoculation. All data are displayed using descriptive analysis.

## Results

### Broth microdilution MIC determination

MIC values were determined for all 131 *K. pneumoniae* and *P. aeruginosa* against the five tested antibiotics (tigecycline tested only against *K. pneumoniae* isolates). Of the total isolate number, 59 isolates meeting the definition of low phenotypic variability were advanced into the *in vivo* model. The number of replicates needed to determine a modal value ranged from 3 to 15. Modal MICs, range of MIC values, and number of broth microdilution replicates used to determine the mode for each isolate introduced into the *in vivo* model are presented for all *K. pneumoniae* and *P. aeruginosa* strains in Tables 1 and 2, respectively. Available genotypic data from the CDC and FDA Antimicrobial Resistance Isolate Bank and the ERACE-PA surveillance programme are provided separately (Tables S1 and S2, available as Supplementary data at *JAC* Online) as well as select isolates that were run on the BioFire^®^ FilmArray^®^ Pneumonia Panel.^13,14^

**Table 1.** MICs determined by broth microdilution against *Klebsiella pneumoniae* isolates tested *in vivo*

		MIC (mg/L)														
Isolate origin	Isolate ID	TGC modal	TGC range	No. of replicates	LVX modal	LVX range	No. of replicates	MEM modal	MEM range	No. of replicates	FDC modal	FDC range	No. of replicates	TOB modal	TOB range	No. of replicates
CAIRD	Kp 266	0.5	^a^	3	>32	^a^	3	4	^a^	3	1	^a^	3	64	64->64	3
CDC Bank	49	4	^a^	4	>32	^a^	3	>64	^a^	3	4	1–8	12	>64	^a^	3
CDC Bank	68	2	^a^	3	>32	^a^	3	>64	32–>64	3	4	^a^	3	>64	^a^	3
CDC Bank	87	2	^a^	3	>32	^a^	3	0.25	≤0.063–0.25	8	4	^a^	3	0.25	0.25–0.5	3
CDC Bank	98	0.5	^a^	3	>32	^a^	3	16	^a^	3	0.5	^a^	3	>64	^a^	3
CDC Bank	106	2	^a^	3	>32	^a^	3	>64	^a^	3	8	4–8	3	>64	^a^	3
CDC Bank	129	1	0.5–1	3	>32	^a^	3	16	^a^	3	2	^a^	3	32	^a^	3
CDC Bank	160	0.25	^a^	3	0.063	0.063–0.125	3	8	8–16	3	0.125	0.125–0.25	3	0.25	0.25–0.5	3
CDC Bank	504	0.5	0.25–0.5	3	0.5	^a^	3	8	^a^	4	0.25	0.25–0.5	3	8	8–16	3
CDC Bank	522	0.5	^a^	3	32	^a^	3	64	^a^	3	8	^a^	3	1	^a^	3
CDC Bank	523	1	^a^	4	4	4–8	4	64	32–64	4	2	^a^	4	16	8–16	4
CDC Bank	542	4	2–4	3	>32	^a^	3	2	^a^	3	>32	^a^	3	>64	^a^	3
CDC Bank	548	2	^a^	3	>32	^a^	3	16	8–16	3	1	1–4	5	0.25	0.25–0.5	3
CDC Bank	550	1	^a^	3	>32	^a^	3	64	64–>64	3	2	^a^	3	32	32–64	3
CDC Bank	553	0.125	0.125–0.5	9	16	8–16	3	2	^a^	3	0.125	^a^	3	>64	^a^	3
CDC Bank	555	2	^a^	3	>32	^a^	3	>64	^a^	3	4	4–8	3	>64	^a^	3
CDC Bank	558	0.5	0.5–1	3	8	8–16	3	2	^a^	3	0.5	0.25–0.5	3	>64	^a^	3
CDC Bank	560	1	1–2	3	>32	^a^	3	>64	^a^	3	16	8–16	3	>64	^a^	3
CDC Bank	831	8	4–8	4	4	^a^	4	≤0.063	^a^	3	0.5	^a^	3	8	^a^	3
CDC Bank	848	4	^a^	3	>32	^a^	3	64	^a^	3	4	^a^	3	>64	^a^	3
CDC Bank	851	0.25	0.25–0.5	3	0.063	^a^	3	≤0.063	^a^	3	0.063	≤0.031–0.25	15	16	^a^	3
CDC Bank	860	2	^a^	3	32	32->32	3	4	4–8	3	4	^a^	3	4	2–4	3
PEI	Kp C1.104	0.5	^a^	3	≤0.063	^a^	3	≤0.063	^a^	3	0.25	0.25–0.5	3	0.5	0.25–0.5	6
PEI	Kp C1.111	0.5	^a^	3	0.063	≤0.063–0.25	5	≤0.063	^a^	3	0.13	0.13–0.25	3	0.25	0.25–0.5	6
PEI	Kp C1.112	0.25	0.25–0.5	3	≤0.063	≤0.063–0.125	3	≤0.063	^a^	3	0.25	0.13–0.25	3	0.5	0.25–0.5	6
PEI	Kp C1.113	0.25	0.25–0.5	3	≤0.063	≤0.063–0.125	3	≤0.063	^a^	3	0.25	0.13–0.25	3	0.25	^a^	6
PEI	Kp C1.147	1	^a^	3	32	32->32	3	32	16–32	3	2	1–2	3	>64	^a^	3
PEI	Kp C1.151	1	^a^	3	>32	^a^	3	>64	^a^	3	2	1–2	3	32	16–32	3
DSMZ	30 104	0.25	^a^	3	0.063	0.063–0.125	3	0.125	<0.063–0.125	3	0.25	^a^	3	0.125	^a^	4

^a^The range is equivalent to the mode as all replicates had the same MIC value.

Kp, *Klebsiella pneumoniae*; TGC, tigecycline; LVX, levofloxacin; MEM, meropenem; FDC, cefiderocol; TOB, tobramycin.

**Table 2.** MICs determined by broth microdilution against *Pseudomonas aeruginosa* isolates tested *in vivo*

		MIC (mg/L)											
Isolate origin	Isolate ID	LVX modal	LVX range	No. of replicates	MEM modal	MEM range	No. of replicates	FDC modal	FDC range	No. of replicates	TOB modal	TOB range	No. of replicates
CDC Bank	111	16	16–32	3	64	^a^	3	0.25	0.25–0.5	3	>64	64–>64	5
CDC Bank	234	8	^a^	3	2	^a^	3	0.5	0.25–0.5	3	64	^a^	5
CDC Bank	250	32	32–>32	3	>64	^a^	3	4	^a^	3	>64	^a^	3
CDC Bank	256	0.5	^a^	3	4	^a^	3	≤0.031	≤0.031–0.063	3	0.5	^a^	3
CDC Bank	272	8	8–16	3	4	2–4	3	0.063	^a^	3	>64	^a^	3
CDC Bank	354	8	^a^	3	4	^a^	3	0.125	0.063–0.125	3	>64	^a^	3
CDC Bank	356	0.5	0.5–1	3	64	64–>64	3	1	1–2	3	32	32–>64	7
CDC Bank	358	2	^a^	3	16	16–32	3	0.063	^a^	3	32	^a^	3
CDC Bank	439	32	^a^	3	32	^a^	3	0.25	0.125–0.25	4	>64	^a^	3
CDC Bank	443	>32	^a^	3	64	32–>64	5	1	0.125–2	13	>64	^a^	3
CDC Bank	458	1	1–2	3	2	0.5–2	6	0.5	0.125–0.5	6	2	^a^	3
CDC Bank	459	8	8–16	3	16	16–32	3	0.063	^a^	3	2	2–8	8
CDC Bank	511	0.5	^a^	3	4	^a^	4	0.063	^a^	4	1	0.5–1	5
CDC Bank	516	0.5	^a^	3	64	^a^	3	1	0.125–2	12	0.5	^a^	5
CDC Bank	767	32	32–>32	3	>64	^a^	3	8	4–8	4	>64	^a^	3
CDC Bank	771	>32	^a^	3	>64	^a^	3	4	1–4	5	>64	^a^	3
PEI	Pa 88198	4	^a^	3	2	^a^	3	0.25	0.125–0.5	12	0.5	0.25–1	8
PEI	Pa 88276	1	^a^	3	1	0.5–4	8	0.5	0.25–4	13	1	^a^	6
PEI	Pa 88342	1	0.5–1	3	1	0.5–4	10	0.5	0.25–1	7	1	0.5–1	6
PEI	Pa 88356	2	1–8	9	32	32–64	3	4	^a^	3	2	1–4	8
PEI	Pa 88826	1	^a^	3	1	1–2	3	0.063	≤0.031–0.063	3	0.5	^a^	6
PEI	Pa 89268	8	^a^	3	1	^a^	3	0.25	0.063–0.25	8	0.5	0.125–0.5	8
DSMZ	DSM50071	1	^a^	3	2	2–4	3	0.125	0.063–0.25	7	2	*	3
CAIRD	PSA INT-2–41	>32	^a^	3	16	^a^	3	16	^a^	4	>64	^a^	5
CAIRD	PSA INT-4–99	32	^a^	3	8	^a^	3	8	8–16	3	>64	^a^	3
CAIRD	PSA INT-4–100	1	0.5–1	3	4	^a^	3	0.125	^a^	3	0.5	^a^	5
CAIRD	PSA INT-5–19	32	^a^	3	>64	^a^	3	0.125	0.125–0.25	3	>64	^a^	5
CAIRD	PSA INT-5-35	2	^a^	3	16	^a^	3	0.5	0.125–0.5	10	0.5	^a^	3
CAIRD	PSA INT-12-18	16	^a^	3	8	^a^	3	1	1–4	5	16	^a^	3
CAIRD	PSA US-4-27	32	^a^	3	64	^a^	3	16	^a^	3	>64	^a^	3

^a^The range is equivalent to the mode as all replicates had the same MIC value.

LVX, levofloxacin; MEM, meropenem; FDC, cefiderocol; TOB, tobramycin.

### Klebsiella pneumoniae *model performance*

**Figure 1.**
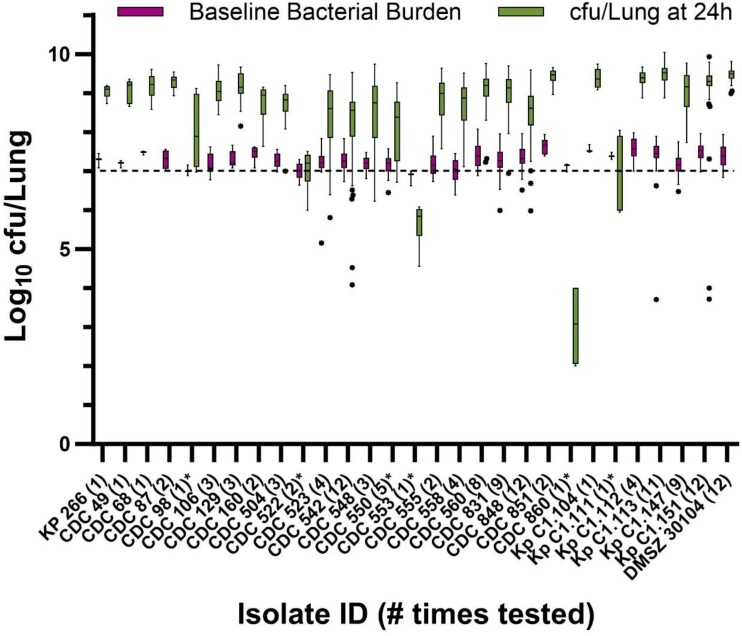
*Klebsiella pneumoniae* baseline bacterial burden and *in vivo* growth in the COMBINE murine neutropenic pneumonia model. The dashed line represents the target baseline burden of 7 log_10_ cfu/lung. Outlier mice were determined by Tukey’s test and are displayed as individual dots. Asterisk denotes isolates unsuitable for the model.

Targeting a baseline bacterial burden of 6–6.5 log_10_ cfu/lung using 16 *K. pneumoniae* isolates, the mean ± SD inoculum, baseline and 24 h were 7.42 ± 0.19 log_10_ cfu/mL, 6.28 ± 0.26 log_10_ cfu/lung and 5.22 ± 1.12 log_10_ cfu/lung, respectively. Only two (13%) (CDC 560 and CDC 106) isolates achieved the requisite mean growth of ≥1 log_10_ cfu/lung for viability in the pneumonia model, albeit with considerable variability (Table S3). Increasing the baseline log_10_ cfu/lung target to 6.5–7 in 11 isolates yielded mean ± SD inoculum, baseline and 24 h of 7.89 ± 0.09 log_10_ cfu/mL, 6.84 ± 0.13 log_10_ cfu/lung and 6.88 ± 1.04 log_10_ cfu/lung, respectively (Table S3). Despite increasing the starting bacterial burdens by 0.5 log, only one (9%) (DSMZ 30104) isolate reached growth of ≥1 log_10_ cfu/lung. Indeed, pushing the starting bacterial burden up to 7–7.5 log_10_ cfu/lung was necessary to consistently achieve viability in the model (Figure 1). Across the 29 isolates studied at the highest inoculum, 25 (83%) reached the minimum 24 h growth threshold and mean ± SD inoculum, baseline and 24 h of 8.40 ± 0.25 log_10_ cfu/mL, 7.29 ± 0.19 log_10_ cfu/lung and 8.45 ± 1.35 log_10_ cfu/lung. Data for individual isolates at each tested inoculum are presented in Table S3. There was negligible (<1%) mortality before scheduled harvest times using these *K. pneumoniae* isolates, regardless of inoculum used. Among the 25 *K. pneumoniae* that met the predefined inclusion criteria for replicate *in vivo* testing, 21 were subsequently tested numerous times (ranging from 2 to 12 replicates). Of note, the other four isolates (Kp 266, CDC 49, CDC 68 and Kp C1.104) performed well in the model when tested in singlet, but had similar phenotypic and genotypic profiles to the other isolates and as such were not explored further. Based on poor or inconsistent growth at 24 h, the following (*n* = 6) isolates were determined unsuitable for use in the COMBINE murine neutropenic pneumonia model: CDC 98, CDC 522, CDC 550, CDC 553, CDC 860 and Kp C1.111.

### Pseudomonas aeruginosa *model performance*

**Figure 2.**
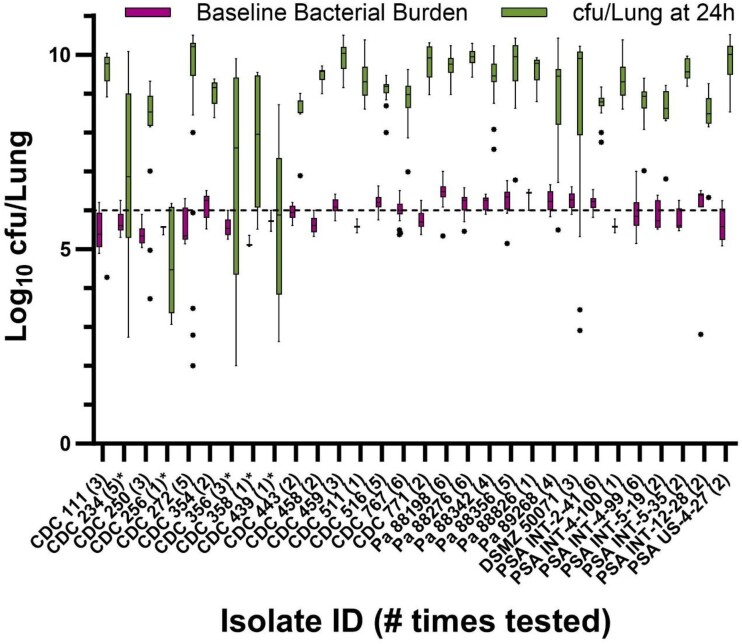
*Pseudomonas aeruginosa* baseline bacterial burden and *in vivo* growth in the COMBINE murine neutropenic pneumonia model. The dashed line represents the target baseline burden of 6 log_10_ cfu/lung. Outlier mice were determined by Tukey’s test and are displayed as individual dots. Asterisk denotes isolates unsuitable for the model.

**Figure 3.**
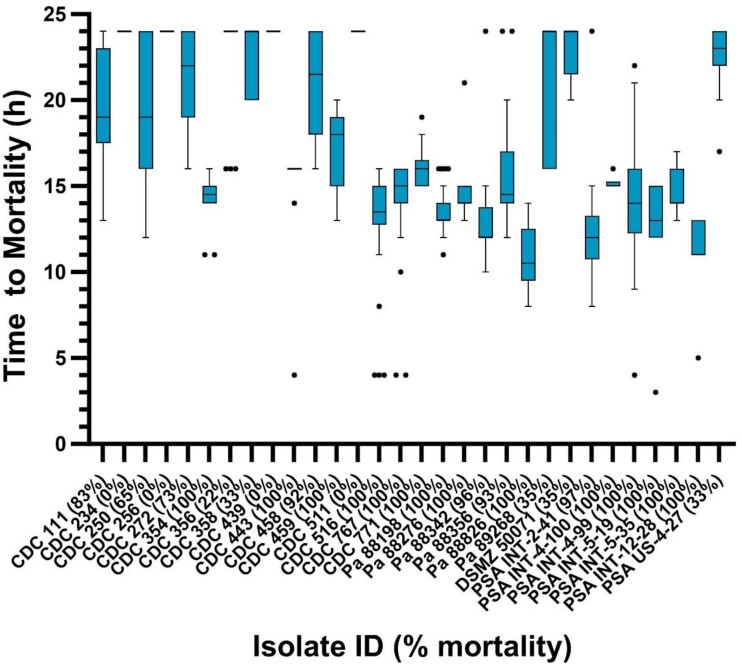
Time to and percentage mortality of *Pseudomonas aeruginosa* isolates in the COMBINE murine neutropenic pneumonia model. Outlier mice were determined by Tukey’s test and are displayed as individual dots. This figure appears in colour in the online version of *JAC* and in black and white in the print version of *JAC*.

Among the 30 isolates tested in the pneumonia model, the mean ± SD inoculum suspension cfu/mL and baseline bacterial burden cfu/lung were 7.49 ± 0.24 and 5.93 ± 0.34, respectively (Figure 2). Most (27/30%–90%) fulfilled the growth threshold of ≥1 log cfu/lung at 24 h. Despite targeting the lower end of starting bacterial burden endorsed in the COMBINE protocol, percentage mortality over the 24 h post-inoculation period was profound with median (IQR) of 96% (35%–100%). Time to mortality across all isolates varied widely with median (IQR) of 16 h (14–24 h) as shown in Figure 3. Inoculum bacterial suspension cfu/mL, baseline bacterial burden cfu/lungs, cfu/lung at 24 h, time to and percentage mortality over the 24 h post-inoculation period for each *P. aeruginosa* isolate tested in the model are shown numerically in the supplement (Table S4). Among the 27 isolates that met the pre-specified criteria for replicate *in vivo* testing, 24 were examined numerous times (ranging from 2–6 replicates). Importantly, the other three isolates (CDC 511, Pa 88826, PSA INT 4–100) performed well in the model during initial *in vivo* assessment, but similarly to some *K. pneumoniae* isolates mentioned previously, had phenotypic and genotypic profiles that were already well represented by other isolates in the cohort and as such were not further explored in the model. Based on poor or inconsistent growth at 24 h, the following (*n* = 5) isolates were determined unsuitable for use in the COMBINE murine neutropenic pneumonia model: CDC 234, CDC 256, CDC 356, CDC 358 and CDC 439.

## Discussion

International harmonization of preclinical PK/PD infection models provides an opportunity to improve clinical translation and inter-laboratory reproducibility. In this current study, our phenotypic profiling efforts were initiated with 131 isolates against five selected reference compounds. Fifty-nine displaying low phenotypic variability were advanced into the *in vivo* environment to determine viability in the COMBINE murine neutropenic pneumonia model. Forty-five of the strains displaying *in vivo* viability in initial studies were subjected to repeat assessment to understand inter-day variability. These 45 isolates encompass a large assemblage of β-lactamases and efflux and porin mutations that will allow for the benchmarking of novel compounds with diverse mechanisms of action/resistance in this *in vivo* model. As this translational model is utilized to evaluate pharmacodynamics, the most important bacterial-derived distinctions for suitable isolates are its phenotypic/genotypic profile, sufficient *in vivo* growth and limited mortality over the first 8 h post-inoculation period where the potential for drug efficacy is unlikely to be recognized due to overwhelming sepsis. While the characterization of other bacterial attributes such as virulence genes/function, sequence types and capsular serotypes may be undertaken, these assessments are not generally done for isolates used in pharmacodynamic models. This translational model and the selection of viable isolates as previously defined is similar to the clinical setting where clinical microbiology laboratories routinely define phenotypic profiles but do not characterize additional bacterial-derived factors such as virulence genes, sequence types and capsular serotypes for the care of patients. Although this additional testing has not been undertaken in the current study, the incorporation of the rate and extent of mortality in the isolate selection process is an assessment of the collective expression of all these factors in the background of the test organisms.

These data provide vital insights into the viability and variability of the COMBINE neutropenic pneumonia model. Importantly, sensitivity to the starting bacterial burden for *K. pneumoniae* isolates was demonstrated, as even half-log differences were shown to improve or impede isolates from establishing viable growth in the model. As such, starting bacterial burdens for *K. pneumoniae* isolates may need to target the upper end or even exceed the COMBINE proposal of 6–7 log_10_ cfu/lung. Conversely, *P. aeruginosa* strains tended to grow well at the lower end of the described starting bacterial burden range. Despite this, mortality was profound before the scheduled harvest time 24 h post-inoculation. Although not directly assessed, starting *P. aeruginosa* isolates at the upper end of the defined COMBINE range could plausibly enhance the rate of mortality such that even PK/PD optimized antibiotic exposures may be unable to produce meaningful reductions in cfu/lung due to enhanced severity of the infection and overwhelming sepsis. Additionally, among the 45 isolates that were tested multiple times (up to 12) *in vivo* on separate experiment days, only six had inter-run categorical differences in reaching 1 log_10_ cfu/lung of net growth at 24 h, underscoring model reproducibility and stability.

This paper lays the groundwork for establishing a benchmark of expected results against a broad array of *K. pneumoniae* and *P. aeruginosa* by robustly describing expected viability and variability of many model elements against key target pathogens inclusive of broth microdilution MICs, required inoculums, initial bacterial burden, growth at 24 h, time to and percentage mortality. With the stability of the test isolates and model now established, further explorations can focus on the use of this translational PK/PD model with the five selected clinically available therapies to provide a foundation suitable for benchmarking novel therapeutic compounds. These derived data in conjunction with the availability of the isolates to investigators via a central repository will serve as a resource for organism selection when using the COMBINE murine pneumonia model methodology. This paper serves to identify suitable isolates for the model, providing the framework as part one of a four-part series describing the activity and variability in the model of human-simulated exposures of both plasma and pulmonary ELF for tigecycline, levofloxacin, meropenem, cefiderocol and tobramycin against a selection of the isolates defined herein. With established clinical use of these compounds for the treatment for Gram-negative pneumonia, humanized exposures in the murine model will allow for back-translation of anticipated efficacy and provide meaningful benchmarks for cfu/lung assessments of novel compounds under development.

## Supplementary Material

dkae388_Supplementary_Data

## Supplementary data

Tables S1 to S4 are available as Supplementary data at *JAC* Online.
