# Supplementary material for: Establishment of a diverse pheno-genotypic challenge set of Klebsiella pneumoniae and Pseudomonas aeruginosa suitable for use in the murine pneumonia model
Source: J Antimicrob Chemother. 2024 Oct 30;80(1):154–61. doi: 10.1093/jac/dkae388 (PMC11695912; doi:10.1093/jac/dkae388)
Supplement: dkae388_Supplementary_Data [file dkae388_supplementary_data.docx]

**Establishment of a Diverse Pheno-Genotypic Challenge Set of *Klebsiella pneumoniae* and *Pseudomonas aeruginosa* Suitable for Use in the Murine Pneumonia Model**

*Journal of Antimicrobial Chemotherapy*

Andrew J. FRATONI, Alissa M. PADGETT, Erin M. DUFFY, David P. NICOLAU

Supplemental Data File

Version 10/3/24

**Table S1.** Known genotypic information for *Klebsiella pneumoniae* isolates

| **Isolate Origin** | **Isolate ID** | **Known Genotypic Information** |
| --- | --- | --- |
| CAIRD | KP 266 | ESBL |
| CDC Bank | 49 | aac(3)-IIa, aac(6')-Ib-D181Y, aadA2, aph(3')-Ib, aph(6)-Id, ble-MBL, catB4, CMY-6, CTX-M-15, dfrA14, EMRD, KDEA, mph(A), NDM-1, Omp35, OmpK35, oqxA, oqxB, OXA-1, qacEdelta1, QnrB9, rmtC, SHV-53, sul1, sul2, TEM-1, tet(A), tet(R) |
| CDC Bank | 68 | aac(3)-IId, aac(6')-Ib, aadA1, aadA2, aph(3")-Ib, aph(3')-VI, aph(6)-Id, armA, ble-MBL, catA1, catB4, CTX-M-15, dfrA1, dfrA12, EMRD, fosA5, KDEA, mph(E), msr(E), NDM-1, oqxA, oqxB20, OXA-1, OXA-232, OXA-9, qacEdelta1, sat2, SHV-11, sul1, sul2, TEM-1A |
| CDC Bank | 87 | EMRD, KDEA, Omp35, OmpK35, oqxA, oqxB20, QnrB1, SHV-12, tet(A), tet(R) |
| CDC Bank | 98 | aac(3)-Iva, aac(6')-Ib, aadA1, aph(4)-Ia, catA1, cmlA1, dfrA12, EMRD, KDEA, KPC-2, mph(A), Omp35, OmpK35, oqxA, oqxB, OXA-9, qacEdelta1, qacL, SHV-12, sul1, sul3, TEM-1A |
| CDC Bank | 106 | aac(3)-IId, aac(6')-Ib, aadA1, aadA2, aph(3")-Ib, aph(3')-VI, aph(6)-Id, armA, ARR-2, ble-MBL, catB4, cmlA5, CTX-M-15, dfrA1, dfrA12, EMRD, ere(A), fosA5, KDEA, mph(E), msr(E), NDM-1, oqxA, oqxB20, OXA-1, OXA-9, qacEdelta1, sat2, SHV-28, sul1, sul2, TEM-1A |
| CDC Bank | 129 | aac(6')-Ib, aadA2, aph(3')-Ia, catA1, dfrA12, KPC-3, mph(A), OmpK35, oqxA, oqxB, sul1, TEM-1A |
| CDC Bank | 160 | fosA, oqxA, oqxB, OXA-48, SHV-11 |
| CDC Bank | 504 | aac(6')-Ib-cr, catB4, CTX-M-15, OXA-48(c), SHV-OSBL(b), tet(A), tet(R) |
| CDC Bank | 522 | aadA2, aph(3')-Ia, dfrA12, KPC-2, mph(A), oqxA, SHV-11, sul1 |
| CDC Bank | 523 | aadA1, aph(3')-Ia, dfrA1, KPC-2, sul1 |
| CDC Bank | 542 | aac(6')-Ib, aadA2, catA1, dfrA12, EMRD, KDEA, mph(A), oqxA, oqxB, SHV-12, sul1 |
| CDC Bank | 548 | aph(3")-Ib, aph(6)-Id, CTX-M-15, dfrA14, EMRD, fosA, KDEA, KPC-3, oqxA, oqxB20, QnrS1, SHV-28, sul2, TEM-1 |
| CDC Bank | 550 | aadA2, ant(2")-la, catA1, dfrA12, EMRD, KDEA, KPC-3, mph(A), oqxA, oqxB, QnrS1, SHV-189, sul1 |
| CDC Bank | 553 | aac(3)-IId, aadA2, armA, CTX-M-15, dfrA12, dfrA14, EMRD, fosA5, KDEA, OXA-181, SHV-26, sul1, sul2 |
| CDC Bank | 555 | aac(6')-Ib-G, aadA1, aph(3')-Ia, ARR-2, ble-MBL, catB, CTX-M-15, dfrA14, EMRD, KDEA, mph(A), NDM-5, oqxA, oqxB25, OXA-232, OXA-9, QnrS1, rmtF1, SHV-12, sul1, TEM-1A |
| CDC Bank | 558 | aac(3)-IId, aac(6')-Ib-cr, aadA1, aadA2, armA, ARR-2, catA1, CTX-M-15, dfrA12, dfrA14, EMRD, ere(A), fosA5, KDEA, OXA-181, SHV-26, sul1, sul2, tet(A), tet(R) |
| CDC Bank | 560 | aac(3)-IId, aac(6')-Ib-AKT, aadA1, armA, ARR-2, ble-MBL, catA1, catB4, cmlA5, CMY-4, CTX-M-15, dfrA1, EMRD, ere(A), fosA, KDEA, mph(E), msr(E), NDM-1, oqxA, oqxB20, OXA-1, OXA-9, sat2, SHV-100, sul1, TEM-1A |
| CDC Bank | 831 | aac(3)-IIa, aph(3')-Ib, aph(6)-Id, catB4, CTX-M-15, dfrA14, OXA-1, QnrB1, SHV-187, sul2, TEM-1B, tet(A), tet(R) |
| CDC Bank | 848 | aac(3)-IIa, aac(6')-Ib-cr, catB4, CTX-M-15, dfrA1, Omp35, OmpK35, oqxA, oqxB, OXA-1, OXA-48, QnrS1, SHV-11, sul1, TEM-1B, tet(A), tet(R) |
| CDC Bank | 851 | SHV-11 |
| CDC Bank | 860 | aph(3')-Ib, aph(6)-Id, catB4, CTX-M-15, dfrA14, Omp35, OmpK35, QnrB1, SHV-1, TEM-1B, tet(A), tet(R) |
| PEI | Kp C1.104 | Unknown |
| PEI | Kp C1.111 | Unknown |
| PEI | Kp C1.112 | Unknown |
| PEI | Kp C1.113 | Unknown |
| PEI | Kp C1.147 | CTX-M, OXA-48 Like* |
| PEI | Kp C1.151 | KPC* |
| DSMZ | 30104 | Unknown |

CAIRD, Center for Anti-Infective Research and Development; CDC, Centers for Disease Control and Prevention; PEI, Paul Ehrlich Institute; DSMZ, Leibniz Institute. *Tested on the BioFire® FilmArray® Pneumonia Panel

**Table S2.** Known genotypic information for *Pseudomonas aeruginosa* isolates

| **Isolate Origin** | **Isolate ID** | **Known Genotypic Information** |
| --- | --- | --- |
| CDC Bank | 111 | aac(3)-Id, aac(6')-Il, aadA2, aph(3')-IIb, bcr1, cmlA6, dfrB5, floR2, fosA, mexA, mexE, mexX, OXA-4, OXA-486, PDC-3, qacEdelta1, qacF, tet(G), VIM-2 |
| CDC Bank | 234 | aac(6')-Ib-G, aadA6, aph(3')-IIb, aph(6)-Id, bcr1, fosA, mexA, mexE, OXA-488, PDC-35, strA, sul1, tet(C) |
| CDC Bank | 250 | aac(6')-Il, aadA1, ant(2")-la, aph(3')-IIb, bcr1, dfrB2, floR2, fosA, mexA, mexE, NDM-1, OXA-10, OXA-846, PDC-11, PME-1, rmtD2, sul1, tet(G), tet(R), VEB-9 |
| CDC Bank | 256 | aph(3')-IIb, bcr1, catB7, fosA, mexA, mexE, mexX, OXA-50, PDC-8 |
| CDC Bank | 272 | aph(3')-IIb, bcr1, catB7, fosA, mexA, mexE, mexX, OXA-395, PDC-5 |
| CDC Bank | 354 | aadA11, ant(2")-la, aph(6)-Id, dfrB1, strA |
| CDC Bank | 356 | KPC-2 |
| CDC Bank | 358 | bcr1, dfrA8 |
| CDC Bank | 439 | aac(6')-Il, aadA1b, bcr1, IMP-18, OXA-50 |
| CDC Bank | 443 | aac(6')-Il, ant(2")-la, OXA-10, tet(R), VEB-1 |
| CDC Bank | 458 | bcr1 |
| CDC Bank | 459 | OXA-50 |
| CDC Bank | 511 | OXA-50 |
| CDC Bank | 516 | KPC-2 |
| CDC Bank | 767 | GES-20 |
| CDC Bank | 771 | GES-19, GES-20 |
| PEI | Pa 88198 | Unknown |
| PEI | Pa 88276 | Unknown |
| PEI | Pa 88342 | Unknown |
| PEI | Pa 88356 | Unknown |
| PEI | Pa 88826 | Unknown |
| PEI | Pa 89268 | Unknown |
| DSMZ | 50071 | Unknown |
| CAIRD | PSA INT-2-41 | aac(6')-Ib,aac(6')-Ib-cr,aadA6,aph(3')-IIb,CTX-M-2,OXA-488,PDC-35,catB7,fosA,qacE,sul1 |
| CAIRD | PSA INT-4-99 | aac(6')-Ib-cr,aadA6,aph(3')-IIb,OXA-14,OXA-488,PDC-35,catB7,cml,cmlA1,fosA,qacE,sul1 |
| CAIRD | PSA INT-4-100 | OXA-395, PDC-123 |
| CAIRD | PSA INT-5-19 | OXA-488, PAO (PDC-35), GES-5 |
| CAIRD | PSA INT-5-35 | aph(3')-IIb,OXA-488,PDC-471,catB7,fosA |
| CAIRD | PSA INT-12-18 | aac(6')-Ib,aadA6,aph(3'')-Ib,aph(3')-IIb,aph(3')-VIb,aph(3')-XV,aph(6)-Id,GES-1,OXA-488,PDC-35,PER-1,catB7,fosA,qacE,sul1,tet(G) |
| CAIRD | PSA US-4-27 | OXA-17/142, OXA-395, PAO (PDC-44), VIM-2 |

CAIRD, Center for Anti-Infective Research and Development; CDC, Centers for Disease Control and Prevention; PEI, Paul Ehrlich Institute; DSMZ, Leibniz Institute.

**Table S3.** *Klebsiella pneumoniae* *in vivo* growth in the COMBINE murine neutropenic lung infection model at different baseline bacterial burden targets. Data are presented as mean ± standard deviation. “-“ denotes not tested.

|  |  | **0h Log_10_ cfu/Lung Target** | | | | | | | | |
| --- | --- | --- | --- | --- | --- | --- | --- | --- | --- | --- |
|  |  | **6-6.5** | | | **6.5-7** | | | **7-7.5** | | |
| **Isolate Origin** | **Isolate ID** | **Inoculum cfu** | **0h cfu/Lung** | **24h cfu/Lung** | **Inoculum cfu** | **0h cfu/Lung** | **24h cfu/Lung** | **Inoculum cfu** | **0h cfu/Lung** | **24h cfu/Lung** |
| CAIRD | KP 266 | - | - | - | 7.83±0.05 | 6.91±0.17 | 5.77±1.07 | 8.55 | 7.28±0.19 | 9.04±0.17 |
| CDC Bank | 49 | 7.60 | 6.14±0.18 | 5.31±0.82 | - | - | - | 8.66 | 7.18±0.09 | 9.05±0.30 |
| CDC Bank | 68 | 7.11 | 6.23±0.39 | 5.21±1.57 | - | - | - | 8.66 | 7.47±0.05 | 9.17±0.35 |
| CDC Bank | 87 | 7.51 | 6.44±0.20 | 4.54±1.12 | - | - | - | 8.51±0.23 | 7.30±0.24 | 8.87±0.18 |
| CDC Bank | 98 | - | - | - | - | - | - | 7.64 | 7.01±0.13 | 7.99±0.96 |
| CDC Bank | 106 | 7.63 | 6.22±0.20 | 7.35±1.40 | 7.95±0.22 | 6.82±0.17 | 6.24±1.39 | 8.31±0.22 | 7.20±0.28 | 9.06±0.35 |
| CDC Bank | 129 | - | - | - | - | - | - | 8.14±0.48 | 7.31±0.20 | 9.15±0.41 |
| CDC Bank | 160 | - | - | - | - | - | - | 7.97* | 7.46±0.18 | 8.69±0.56 |
| CDC Bank | 504 | - | - | - | - | - | - | 8.25±0.13 | 7.28±0.18 | 8.66±0.53 |
| CDC Bank | 522 | 7.31 | 6.15±0.10 | 4.07±0.08 | - | - | - | 8.73±0.07 | 7.00±0.22 | 7.05±0.47 |
| CDC Bank | 523 | 7.29 | 6.43±0.11 | 5.61±1.48 | - | - | - | 8.28±0.21 | 7.16±0.54 | 8.38±0.92 |
| CDC Bank | 542 | 7.42 | 6.53±0.11 | 4.21±0.22 | 7.83 | 6.73±0.23 | 5.90±1.77 | 8.35±0.23 | 7.27±0.24 | 8.20±1.03 |
| CDC Bank | 548 | - | - | - | - | - | - | 8.26±0.18 | 7.16±0.20 | 8.39±1.09 |
| CDC Bank | 550 | 7.14 | 6.36±0.13 | 4.71±0.88 | - | - | - | 8.09±0.17 | 7.14±0.26 | 8.14±0.81 |
| CDC Bank | 553 | 7.64 | 5.94±0.51 | 4.33±0.61 | - | - | - | 8.76 | 6.85±0.20 | 5.65±0.56 |
| CDC Bank | 555 | 7.42 | 6.32±0.24 | 5.59±1.88 | - | - | - | 8.38±0.22 | 7.19±0.59 | 8.82±0.59 |
| CDC Bank | 558 | 7.23 | 6.43±0.35 | 4.22±0.39 | - | - | - | 8.32±0.26 | 7.00±0.34 | 8.66±0.62 |
| CDC Bank | 560 | 7.55 | 6.51±0.27 | 7.71±1.68 | 7.97±0.04 | 6.86±0.32 | 6.44±1.92 | 8.35±0.24 | 7.39±0.30 | 9.07±0.49 |
| CDC Bank | 831 | 7.34 | 6.46±0.20 | 5.89±1.61 | 7.93 | 6.90±0.16 | 7.01±0.96 | 8.41±0.23 | 7.27±0.35 | 9.01±0.48 |
| CDC Bank | 848 | 7.74 | 6.59±0.29 | 4.71±1.22 | 7.95 | 6.72±0.26 | 6.32±1.21 | 8.41±0.24 | 7.35±0.28 | 8.48±0.65 |
| CDC Bank | 851 | 7.57 | 6.19±0.26 | 6.07±1.21 | - | - | - | 8.77±0.10 | 7.64±0.21 | 9.41±0.21 |
| CDC Bank | 860 | 7.29 | 5.54±0.32 | 4.00±0.00 | - | - | - | 8.67 | 7.10±0.10 | 3.04±1.06 |
| PEI | Kp C1.104 | - | - | - | 7.81±0.21 | 6.97±0.30 | 7.82±0.75 | 8.56 | 7.55±0.11 | 9.38±0.25 |
| PEI | Kp C1.111 | - | - | - | - | - | - | 8.41 | 7.39±0.09 | 6.97±0.88 |
| PEI | Kp C1.112 | - | - | - | 7.82±0.08 | 7.04±0.11 | 8.03±1.12 | 8.56±0.27 | 7.57±0.32 | 9.39±0.20 |
| PEI | Kp C1.113 | - | - | - | - | - | - | 8.36±0.24 | 7.40±0.53 | 9.50±0.25 |
| PEI | Kp C1.147 | - | - | - | 7.96 | 6.88±0.34 | 5.83±1.55 | 8.33±0.27 | 7.16±0.27 | 9.09±0.47 |
| PEI | Kp C1.151 | - | - | - | 8.00±0.30 | 6.87±0.46 | 7.38±1.12 | 8.40±0.17 | 7.50±0.21 | 9.13±0.99 |
| DSMZ | 30104 | - | - | - | 7.73±0.03 | 6.54±0.85 | 8.94±0.92 | 8.22±0.24 | 7.39±0.28 | 9.47±0.16 |

*****Error in the serial dilution and plating of the inoculum on one of the test dates, therefore no SD reported. CAIRD, Center for Anti-Infective Research and Development; CDC, Centers for Disease Control and Prevention; PEI, Paul Ehrlich Institute; DSMZ, Leibniz Institute.

**Table S4.** *Pseudomonas aeruginosa* *in vivo* growth and mortality in the COMBINE murine neutropenic lung infection model.

|  |  |  |  |  | **Mortality** | | |
| --- | --- | --- | --- | --- | --- | --- | --- |
| **Isolate Origin** | **Isolate ID** | **Inoculum cfu**  **(mean±SD)** | **0h cfu/Lung**  **(mean±SD)** | **24 cfu/Lung**  **(mean±SD)** | **Median (h)** | **Range (h)** | **%** |
| CDC Bank | 111 | 7.53±0.30 | 5.46±0.47 | 9.36±1.31 | 19 | 13-24 | 83% |
| CDC Bank | 234 | 7.37±0.12 | 5.70±0.29 | 7.06±2.16 | 24 | 24-24 | 0% |
| CDC Bank | 250 | 7.53±0.29 | 5.37±0.27 | 8.07±1.51 | 19 | 12-24 | 65% |
| CDC Bank | 256 | 7.28 | 5.50±0.12 | 4.61±1.32 | 24 | 24-24 | 0% |
| CDC Bank | 272 | 7.50±0.28 | 5.57±0.42 | 9.16±2.38 | 22 | 16-24 | 73% |
| CDC Bank | 354 | 7.39±0.16 | 6.13±0.34 | 9.03±0.32 | 14.5 | 11-16 | 100% |
| CDC Bank | 356 | 7.36±0.22 | 5.57±0.23 | 6.87±2.76 | 24 | 16-24 | 22% |
| CDC Bank | 358 | 7.18 | 5.18±0.16 | 7.78±1.85 | 24 | 20-24 | 33% |
| CDC Bank | 439 | 7.61 | 5.73±0.27 | 5.70±2.10 | 24 | 24-24 | 0% |
| CDC Bank | 443 | 7.46±0.01 | 5.94±0.19 | 8.57±0.58 | 16 | 4-16 | 100% |
| CDC Bank | 458 | 6.72±0.14 | 5.63±0.24 | 9.49±0.21 | 21.5 | 16-24 | 92% |
| CDC Bank | 459 | 7.44±0.31 | 6.11±0.18 | 9.90±0.42 | 18 | 13-20 | 100% |
| CDC Bank | 511 | 7.62 | 6.08±0.21 | 6.97±1.72 | 24 | 24-24 | 0% |
| CDC Bank | 516 | 7.50±0.14 | 6.22±0.21 | 9.10±0.29 | 13.5 | 4-16 | 100% |
| CDC Bank | 767 | 7.37±0.08 | 5.98±0.29 | 8.98±0.53 | 15 | 4-16 | 100% |
| CDC Bank | 771 | 7.05 | 6.05±0.36 | 8.46±1.49 | 16 | 4-16 | 100% |
| PEI | Pa 88198 | 7.78±0.15 | 6.47±0.30 | 9.73±0.27 | 13 | 11-16 | 100% |
| PEI | Pa 88276 | 7.68±0.25 | 6.20±0.25 | 9.93±0.22 | 14 | 13-21 | 100% |
| PEI | Pa 88342 | 7.75±0.36 | 6.18±0.17 | 9.37±0.59 | 12 | 10-24 | 96% |
| PEI | Pa 88356 | 7.54±0.24 | 6.27±0.33 | 9.70±0.75 | 14.5 | 12-24 | 93% |
| PEI | Pa 88826 | 7.88 | 6.33±0.29 | 9.61±0.42 | 10.5 | 8-14 | 100% |
| PEI | Pa 89268 | 7.46±0.05 | 6.25±0.23 | 8.99±1.16 | 24 | 16-24 | 35% |
| DSMZ | 50071 | 7.86±0.24 | 6.25±0.22 | 8.68±2.49 | 24 | 20-24 | 35% |
| CAIRD | PSA INT-2-41 | 7.45±0.26 | 6.20±0.18 | 8.76±0.29 | 12 | 8-24 | 97% |
| CAIRD | PSA INT-4-99 | 7.56±0.17 | 5.89±0.41 | 8.83±0.44 | 14 | 4-22 | 100% |
| CAIRD | PSA INT-4-100 | 7.31 | 5.59±0.18 | 9.35±0.59 | 15 | 15-16 | 100% |
| CAIRD | PSA INT-5-19 | 7.48±0.45 | 5.89±0.35 | 8.58±0.66 | 13 | 3-15 | 100% |
| CAIRD | PSA INT-5-35 | 7.55±0.27 | 5.76±0.29 | 9.60±0.26 | 14 | 13-17 | 100% |
| CAIRD | PSA INT-12-28 | 7.59±0.32 | 5.92±1.18 | 8.37±0.80 | 11 | 11-13 | 100% |
| CAIRD | PSA US-4-27 | 7.43±0.18 | 5.62±0.41 | 9.80±0.57 | 23 | 17-24 | 67% |

CAIRD, Center for Anti-Infective Research and Development; CDC, Centers for Disease Control and Prevention; PEI, Paul Ehrlich Institute; DSMZ, Leibniz Institute.
